# 

**Published:** 1890-03

**Authors:** 


### To Advertisers.

We shall send out, every month, "Journals" to the drug trade, and expect, in this way, to reach a class which will also benefit our advertisers. It will at once be seen that increased profit must accrue to advertisers whose articles are not only introduced to a large medical patronage, but also receive the attention of EVERY DRUGGIST in our territory.

